# Discovery of the Elusive UDP-Diacylglucosamine Hydrolase in the Lipid A Biosynthetic Pathway in *Chlamydia trachomatis*

**DOI:** 10.1128/mBio.00090-16

**Published:** 2016-03-22

**Authors:** Hayley E. Young, Jinshi Zhao, Jeffrey R. Barker, Ziqiang Guan, Raphael H. Valdivia, Pei Zhou

**Affiliations:** a Department of Biochemistry, Duke University Medical Center, Durham, North Carolina, USA; b Department of Molecular Genetics and Microbiology, Duke University Medical Center, Durham, North Carolina, USA

## Abstract

Constitutive biosynthesis of lipid A via the Raetz pathway is essential for the viability and fitness of Gram-negative bacteria, including *Chlamydia trachomatis*. Although nearly all of the enzymes in the lipid A biosynthetic pathway are highly conserved across Gram-negative bacteria, the cleavage of the pyrophosphate group of UDP-2,3-diacyl-GlcN (UDP-DAGn) to form lipid X is carried out by two unrelated enzymes: LpxH in beta- and gammaproteobacteria and LpxI in alphaproteobacteria. The intracellular pathogen *C. trachomatis* lacks an ortholog for either of these two enzymes, and yet, it synthesizes lipid A and exhibits conservation of genes encoding other lipid A enzymes. Employing a complementation screen against a *C. trachomatis* genomic library using a conditional-lethal *lpxH* mutant *Escherichia coli* strain, we have identified an open reading frame (Ct461, renamed *lpxG*) encoding a previously uncharacterized enzyme that complements the UDP-DAGn hydrolase function in *E. coli* and catalyzes the conversion of UDP-DAGn to lipid X *in vitro*. LpxG shows little sequence similarity to either LpxH or LpxI, highlighting LpxG as the founding member of a third class of UDP-DAGn hydrolases. Overexpression of LpxG results in toxic accumulation of lipid X and profoundly reduces the infectivity of *C. trachomatis*, validating LpxG as the long-sought-after UDP-DAGn pyrophosphatase in this prominent human pathogen. The complementation approach presented here overcomes the lack of suitable genetic tools for *C. trachomatis* and should be broadly applicable for the functional characterization of other essential *C. trachomatis* genes*.*

## IMPORTANCE

*Chlamydia trachomatis* is a leading cause of infectious blindness and sexually transmitted disease. Due to the lack of robust genetic tools, the functions of many *Chlamydia* genes remain uncharacterized, including the essential gene encoding the UDP-DAGn pyrophosphatase activity for the biosynthesis of lipid A, the membrane anchor of lipooligosaccharide and the predominant lipid species of the outer leaflet of the bacterial outer membrane. We designed a complementation screen against the *C. trachomatis* genomic library using a conditional-lethal mutant of *E. coli* and identified the missing essential gene in the lipid A biosynthetic pathway, which we designated *lpxG*. We show that LpxG is a member of the calcineurin-like phosphatases and displays robust UDP-DAGn pyrophosphatase activity *in vitro*. Overexpression of LpxG in *C. trachomatis* leads to the accumulation of the predicted lipid intermediate and reduces bacterial infectivity, validating the *in vivo* function of LpxG and highlighting the importance of regulated lipid A biosynthesis in *C. trachomatis*.

## INTRODUCTION

The obligate intracellular bacterium *Chlamydia trachomatis* infects the epithelium of the genital tract and conjunctivae to cause a wide range of diseases, including trachoma, conjunctivitis, salpingitis, pelvic inflammatory disease (PID), and infertility (1). Approximately 6 million people are blinded each year by *C. trachomatis* ocular infections (trachoma), making it the leading cause of infectious blindness worldwide (2). *C. trachomatis* exhibits a biphasic life cycle comprised of two morphological forms: the elementary body (EB) and the reticulate body (RB) (3). *Chlamydia* infection begins when bacteria in the EB form attach to and invade host epithelial cells. The bacteria then differentiate into the RB form and replicate via binary fission within a membrane-bound, pathogen-containing vacuole. In the late stage of infection, bacteria in the RB form differentiate back into the infectious EB form, which is released through cell lysis or extrusion to initiate new rounds of infection.

**FIG 1:**
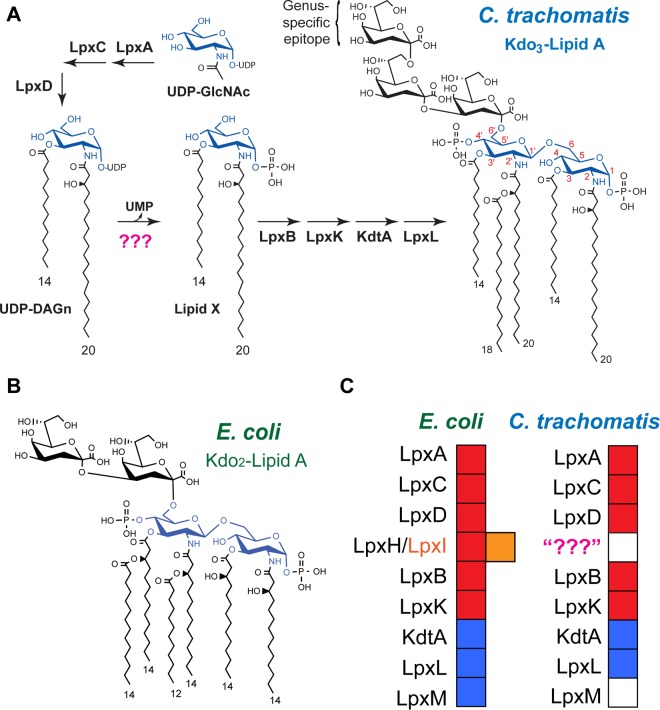
Lipid A and its biosynthetic enzymes. (A) Predicted lipid A biosynthetic pathway (Raetz pathway) in *C. trachomatis*. (B) Structure of *E. coli* Kdo_2_-lipid A. (C) Conservation of lipid A biosynthetic enzymes in *E. coli* and *C. trachomatis*. Essential enzymes are marked by red blocks, nonessential enzymes by blue blocks, and the absence of an enzyme in the genome of *C. trachomatis* is shown by a white block. The existence of an LpxH functional ortholog, LpxI, in alphaproteobacteria is denoted by the orange block next to LpxH.

Like other Gram-negative bacteria, *C. trachomatis* has a dual-membrane cell envelope. Its outer leaflet is composed of lipooligosaccharide (LOS) that displays a short, branching core of three 3-deoxy-d-manno-octulosonic acid (Kdo) residues attached to the hydrophobic membrane anchor, lipid A (Fig. 1A). The *C. trachomatis* lipid A is similar to that in *Escherichia coli* (Fig. 1B), albeit it is penta-acylated, displays longer acyl chains at the 2, 2′, and 2′-3-OH positions, and lacks 3-hydroxyl moieties on the 3 and 3′ chains (4). Like *E. coli* and other Gram-negative species, *C. trachomatis* utilizes enzymes of the Raetz pathway to synthesize lipid A, and nearly all lipid A enzymes have been identified in *C. trachomatis* (Fig. 1C), with the exception of the enzyme responsible for the hydrolysis of UDP-2,3-diacylglucosamine (UDP-DAGn) to lipid X at the fourth step of the pathway. The conversion of UDP-DAGn to lipid X is carried out by either LpxH in beta- and gammaproteobacteria or LpxI in alphaproteobacteria; LpxH and LpxI are unrelated to each other in sequence or catalytic mechanism (5, 6). Neither LpxH nor LpxI is found in *C. trachomatis*, leaving a critical gap in the knowledge of lipid A biosynthetic genes in this prominent human pathogen (Fig. 1C).

Due to a lack of robust tools for genetic manipulation in *Chlamydiae*, the identification and functional characterization of *Chlamydia* gene products has presented unique challenges. To overcome this obstacle and identify the elusive UDP-DAGn hydrolase in *C. trachomatis*, we developed a genetic complementation screen in an *E. coli* strain engineered to have temperature-controlled expression of *lpxH.* Using a *C. trachomatis* expression library, we identified a previously uncharacterized open reading frame (ORF)—*C. trachomatis* ORF 461 (*ct461*), renamed *lpxG*—that is conserved in all *Chlamydia* species. LpxG exhibits robust UDP-DAGn hydrolase activity *in vitro* and *in vivo.* Overexpression of LpxG resulted in the accumulation of lipid X in *C. trachomatis* and profoundly reduced bacterial infectivity, validating LpxG as a novel member of the UDP-DAGn hydrolases and highlighting the importance of regulated lipid A biosynthesis in *Chlamydia* pathogenicity. We suggest that the approach presented in this study may be widely applicable for functional screening of uncharacterized essential genes in *C. trachomatis.*

## RESULTS

### Identification of the *Chlamydia* UDP-DAGn hydrolase through a genetic complementation screen in *E. coli.*

**FIG 2:**
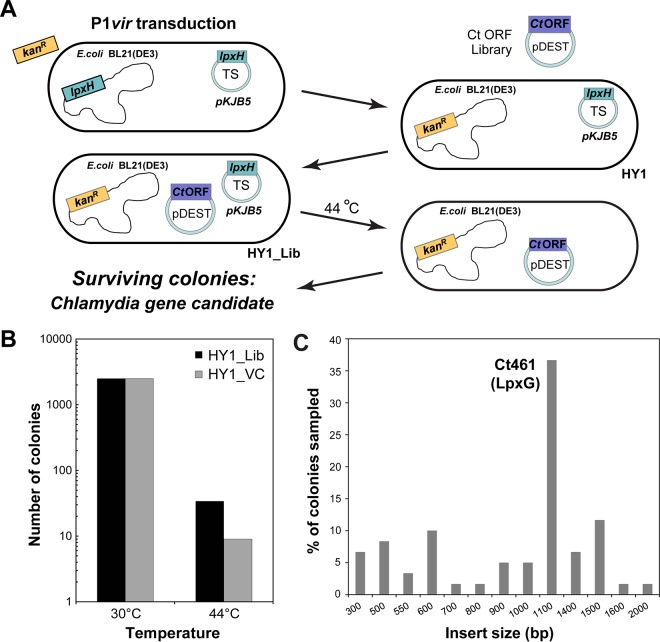
Identification of the *C. trachomatis* UDP-DAGn pyrophosphatase, LpxG. (A) Schematic illustration of the conditional complementation screen. (B) Plating efficiencies of HY1 cells transformed with pDEST (HY1_Lib) or plasmid vector alone (HY1_VC) at permissive (30°C) and restrictive temperatures (44°C). The increase in the number of surviving colonies for HY1_Lib over the number for HY1_VC at 44°C reflects potential complementation by *Chlamydia* gene(s) for the loss of *lpxH* in *E. coli*. (C) Size distribution of the PCR products obtained from surviving colonies with primers spanning the DNA inserts within pDEST.

To identify the gene encoding the UDP-DAGn hydrolase activity in the lipid A biosynthetic pathway of *C. trachomatis*, we devised a temperature-sensitive genetic complementation screen in *E. coli* (Fig. 2A). The temperature-sensitive plasmid pKJB5 containing *E. coli lpxH* was transformed into *E. coli* BL21(DE3), and the chromosomal copy of *lpxH* in these transformed cells was replaced with a *ΔlpxH*::*kan* cassette from *E. coli* strain W3110AΔHEc (6) by P1*vir*-mediated transduction to generate *E. coli* strain HY1. We reasoned that since *lpxH* is an essential gene in *E. coli*, depletion of the temperature-sensitive plasmid at 44°C will be lethal unless the LpxH UDP-DAGn hydrolase activity is provided by a functionally equivalent *Chlamydia* gene product.

We constructed a *C. trachomatis* expression library from a collection of *E. coli* clones harboring all *C. trachomatis* ORFs (ORFome) in pDONR221 (7), which were transferred *en masse* to pDEST17 to place the expression of the inserted *C. trachomatis* DNA under the control of a T7 promoter and a 5′-end ribosomal binding site*.* HY1 cells were transformed with this *C. trachomatis* expression library to generate a collection of expression strains, which we will refer to as HY1_Lib. The equivalent of ~25,000 clones of HY1_Lib were plated and incubated at 44°C. In parallel, HY1 cells were transformed with an empty vector (HY1_VC) to account for the background emergence of spontaneous temperature-resistant HY1 variants. Temperature-resistant clones arose in HY1_Lib at an ~5-fold-greater rate than in HY1 cells transformed with the empty vector (Fig. 2B), suggesting that a factor within the *C. trachomatis* expression library complemented for the loss of the UDP-DAGn hydrolase activity.

To identify the *Chlamydia* gene that complemented an *lpxH* deletion, we first used PCR to determine the size of the *Chlamydia* ORF(s) harbored by the plasmids in temperature-resistant *E. coli* HY1 clones. Nearly 40% of the HY1_Lib colonies surviving at 44°C contained an insert of ~1,100 bp in pDEST17 (Fig. 2C). DNA sequencing of the 1,100-bp PCR product revealed that these plasmids contained *ct461*, which was renamed *lpxG*.

### The *Chlamydia* UDP-DAGn pyrophosphatase LpxG is a Mn^2+^-dependent, membrane-associated calcineurin-like phosphoesterase enzyme.

**FIG 3:**
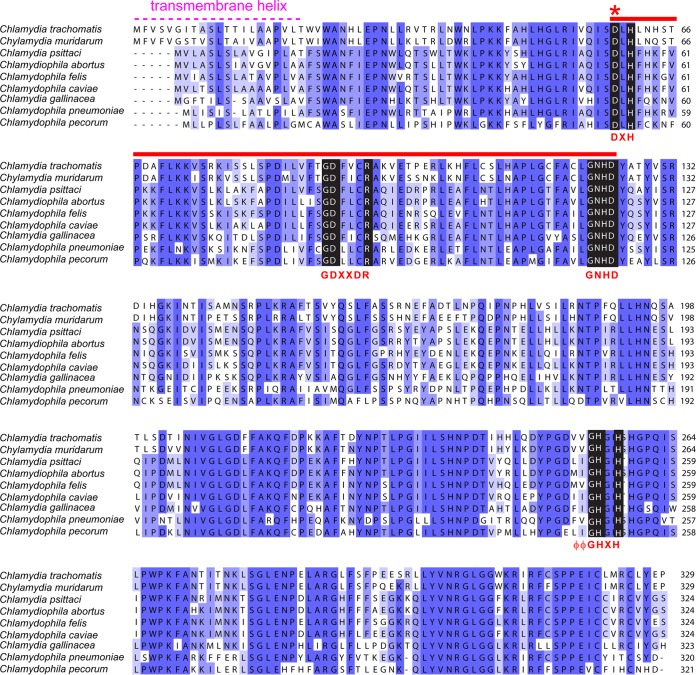
Sequence alignment of LpxG orthologs. Listed are the LpxG orthologs found in the *Chlamydiaceae* family. Residues are colored according to percentage of identity, with darkness of shade corresponding to degree of conservation. CLP motifs are marked in black, with the key amino acid patterns highlighted in red. “X” represents any amino acid, and “ϕ” denotes any hydrophobic residue. The DXH(X)_~25_GDXXDR(X)_~25_GNHD motif is indicated by a red bar, and the location of the predicted transmembrane helix in the *Chlamydia* LpxG is indicated in pink. Sequences were obtained from the NCBI server, and alignment was carried out using ClustalW (32).

The *Chlamydia lpxG* gene encodes a 37-kDa uncharacterized putative metallophosphoesterase. The full-length protein is predicted to have an N-terminal transmembrane helix (Fig. 3), which was truncated from the ORF during construction of the original *C. trachomatis* library (7). We verified that the UDP-DAGn hydrolase activity of LpxH in *E. coli* was also complemented by full-length LpxG, as cells expressing full-length LpxG remained viable upon P1*vir*-mediated transduction of a *ΔlpxH*::*kan* cassette (data not shown).

A protein-protein BLAST search uncovered LpxG orthologs in other *Chlamydia* species (Fig. 3). Alignments of these sequences revealed a DXH(X)_~25_GDXXDR(X)_~25_GNHD motif (with X indicating any amino acid) that is characteristic of enzymes in the calcineurin-like phosphoesterase (CLP) family (8–10). Proteins with this classification utilize a cluster of two to three metal ions to facilitate hydrolysis, with residues from this conserved motif coordinating the metal cofactors (8). Additionally, LpxG possesses a ϕϕGHXH motif (with ϕ representing hydrophobic residues) found in a subclass of CLP enzymes (Fig. 3) (11, 12).

**FIG 4:**
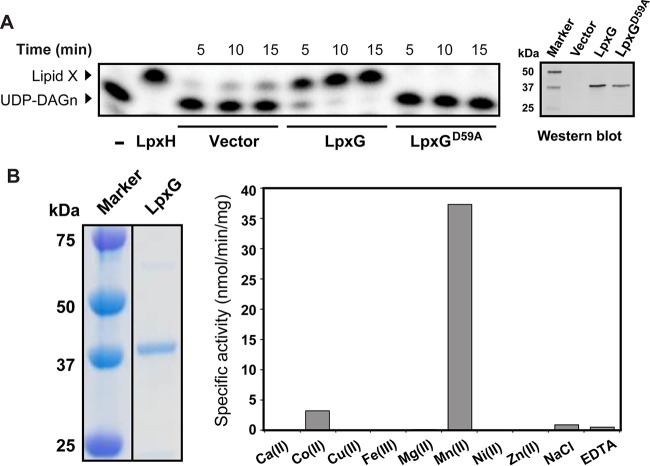
Characterization of the LpxG UDP-DAGn pyrophosphatase activity *in vitro.* (A) Conversion of UDP-DAGn to lipid X by LpxG. Time-dependent accumulation of lipid X is shown for cell-free extracts (CFEs) of cells transformed with an empty vector or a vector expressing LpxG or an LpxG^D59A^ mutant. The total protein concentration in each reaction mixture was ~1 mg/ml. Reaction mixtures with no added enzyme or with purified *Haemophilus influenzae* LpxH were included as negative and positive controls, respectively. Bands corresponding to UDP-DAGn substrate and lipid X product are denoted. The presence of wild-type and mutant LpxG protein in the CFEs was confirmed by immunoblot analysis. (B) Metal dependence of LpxG. Left, LpxG purified by Ni-NTA affinity chromatography. Right, specific activity of LpxG pretreated with EDTA was assayed under standard conditions in reaction buffer supplemented with various divalent or trivalent metal ions, NaCl, or EDTA.

To characterize the *in vitro* activity of LpxG, we expressed C-terminally His_10_-tagged LpxG and an LpxG^D59A^ variant (indicated by an asterisk in Fig. 3) that contained an alanine substitution for the first aspartate residue in the DXH(X)_~25_GDXXDR(X)_~25_GNHD motif, predicted to be required for metal chelating and efficient catalysis of CLP enzymes (8–10). After isopropyl-β-d-thiogalactopyranoside (IPTG) induction, cell-free extracts (CFEs) were prepared from *E. coli* C41(DE3) cells expressing either LpxG or LpxG^D59A^ or cells harboring an empty vector. The extracts were then tested for lipid X production from UDP-DAGn using a thin-layer chromatography (TLC)-based radiographic assay in the presence of a mixture of unlabeled and β-^32^P-labeled substrate. A significantly higher rate of lipid X accumulation was observed in cell extracts from cells expressing LpxG than in an equal amount of extracts prepared from the vector control strain (Fig. 4A). The lipid product generated in the LpxG reactions has a migration property similar to that of the lipid product generated with purified *Haemophilus influenzae* LpxH (13), supporting its identity as lipid X. In contrast, the CFE from cells expressing LpxG^D59A^ displayed little activity, with a level of UDP-DAGn hydrolysis similar to that seen for vector control samples despite a significant level of protein expression, suggesting that the D59A substitution in the predicted metal-chelating motif significantly compromised the catalytic activity of LpxG.

As our *in vitro* assay with CFE of cells expressing LpxG verified an important catalytic residue in the predicted metal-chelating motif of the CLP family of enzymes, we next examined the metal dependency of LpxG using Ni^2+^-NTA–purified enzyme in the presence of detergent (Fig. 4B). The purified enzyme was preincubated with EDTA to remove any copurifying metals. LpxG was then diluted into reaction buffer containing one of the following chloride salts at 1 mM: Ca^2+^, Co^2+^, Cu^2+^, Fe^3+^, Mg^2+^, Mn^2+^, Ni^2+^, or Zn^2+^. Assay conditions where the di- or trivalent metal salt was replaced with 2 mM NaCl or no metal (i.e., in the presence of 1 mM EDTA) were also included as controls. The Mn^2+^ assay condition showed activity that was 35-fold greater than that of the identical reaction with no metal (Fig. 4B). Co^2+^ also enhanced activity, albeit at lower levels (3-fold). The other metal cofactors had little effect on activity. The activity of the enzyme was unaffected by the presence of NaCl, indicating that the ionic strength of the reaction was not responsible for the observed metal stimulation. Overall, our results indicated that Mn^2+^ was the most efficient and thus the most likely cofactor for LpxG activity.

### LpxG hydrolyzes the α-phosphorus atom of UDP-DAGn.

**FIG 5:**
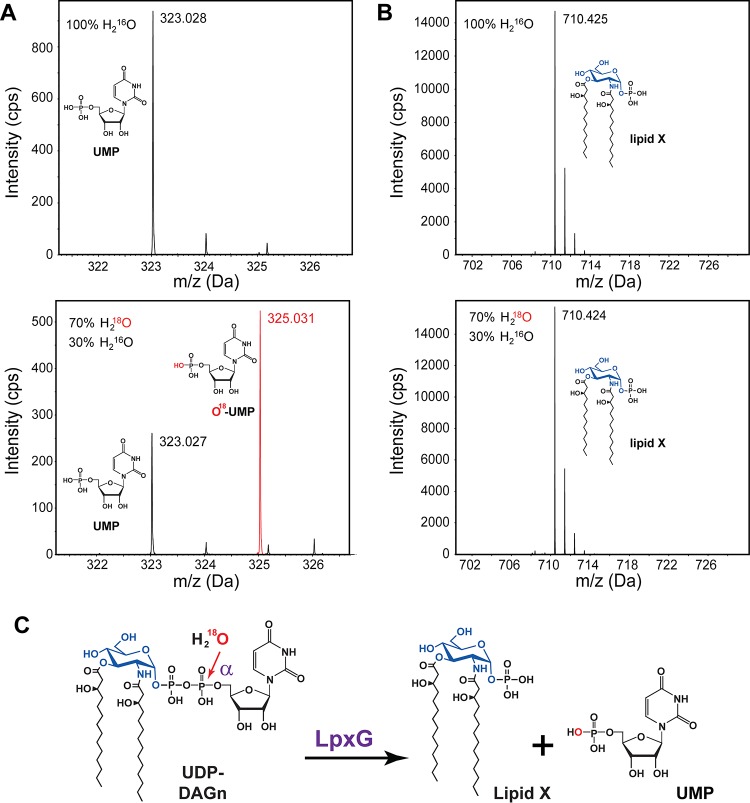
LpxG catalyzes the hydrolysis of UDP-DAGn by attacking the α-phosphate group of UDP. An LpxG-catalyzed UDP-DAGn hydrolysis reaction was carried out in the presence of 70% H_2_^18^O. The results were analyzed by LC-MS and compared with those obtained in the presence of 100% H_2_^16^O. (A) UMP generated by LpxG in the presence of 100% H_2_^16^O was detectable at *m*/*z* 323.028 (top). This peak was also present in the isotopically labeled reaction mixture (bottom); however, a second peak corresponding to the incorporation of ^18^O into UMP was also observed at *m*/*z* 325.031 (red trace). (B) Lipid X generated by LpxG appeared at the same *m*/*z* value in both labeled (top, *m*/*z* 710.425) and unlabeled (bottom, *m*/*z* 710.424) reaction mixtures. (C) Schematic illustration of the LpxG catalysis and incorporation of ^18^O into UMP but not lipid X.

While the other two known UDP-DAGn hydrolases, LpxH and LpxI, both catalyze the formation of lipid X, they do so through different mechanisms: LpxH attacks the α-phosphate of the substrate (5), whereas LpxI attacks at the β position (6). To examine the mechanism employed by LpxG, purified enzyme was used to convert UDP-DAGn to lipid X in the presence of H_2_^16^O and a mixture of H_2_^18^O-H_2_^16^O (70:30, vol/vol). The lipid X and UMP products were extracted using an acidic single-phase Bligh-Dyer system (14), as was reported previously (6). A sample of the LpxG reaction extraction product was analyzed by reverse-phase liquid chromatography coupled with electrospray ionization/mass spectrometry (LC-ESI/MS) operated in the negative ion mode. The UMP product, with a predicted [M−H]^−^ ion at *m*/*z* 323.029, was detected at *m*/*z* 323.028 (Fig. 5A). For the H_2_^18^O-labeled reaction mixture, ~70% of the UMP product contained ^18^O, as shown by the presence of a much more intense peak at *m*/*z* 325.031 versus that at *m*/*z* 323.027 (Fig. 5A, bottom). The lipid X product, predicted to have a [M−H]^−^ ion at *m*/*z* 710.425, was also detectable at the predicted *m*/*z*. In this case, no mass shift in the lipid X peak was observed in the product of the reaction carried out in the presence of H_2_^18^O (Fig. 5B). Taken together, our observations indicate that LpxG catalyzes the attack of water exclusively on the α-phosphorus atom of UDP-DAGn and incorporates the solvent-derived oxygen atom into UMP instead of lipid X (Fig. 5C).

### Overexpression of LpxG in *C. trachomatis* leads to accumulation of chlamydial lipid X and a loss of bacterial infectivity.

To determine whether LpxG is a *bona fide* UDP-DAGn hydrolase, we examined the consequence of LpxG overexpression in *C. trachomatis*. Since the overexpression of many lipid A enzymes is toxic in *E. coli* due to the accumulation of lipid A intermediates (15), we predicted that overexpression of LpxG but not of the inactive LpxG^D59A^ mutant should similarly cause buildup of lipid X and bacterial toxicity, resulting in reduced *Chlamydia* infectivity.

**FIG 6:**
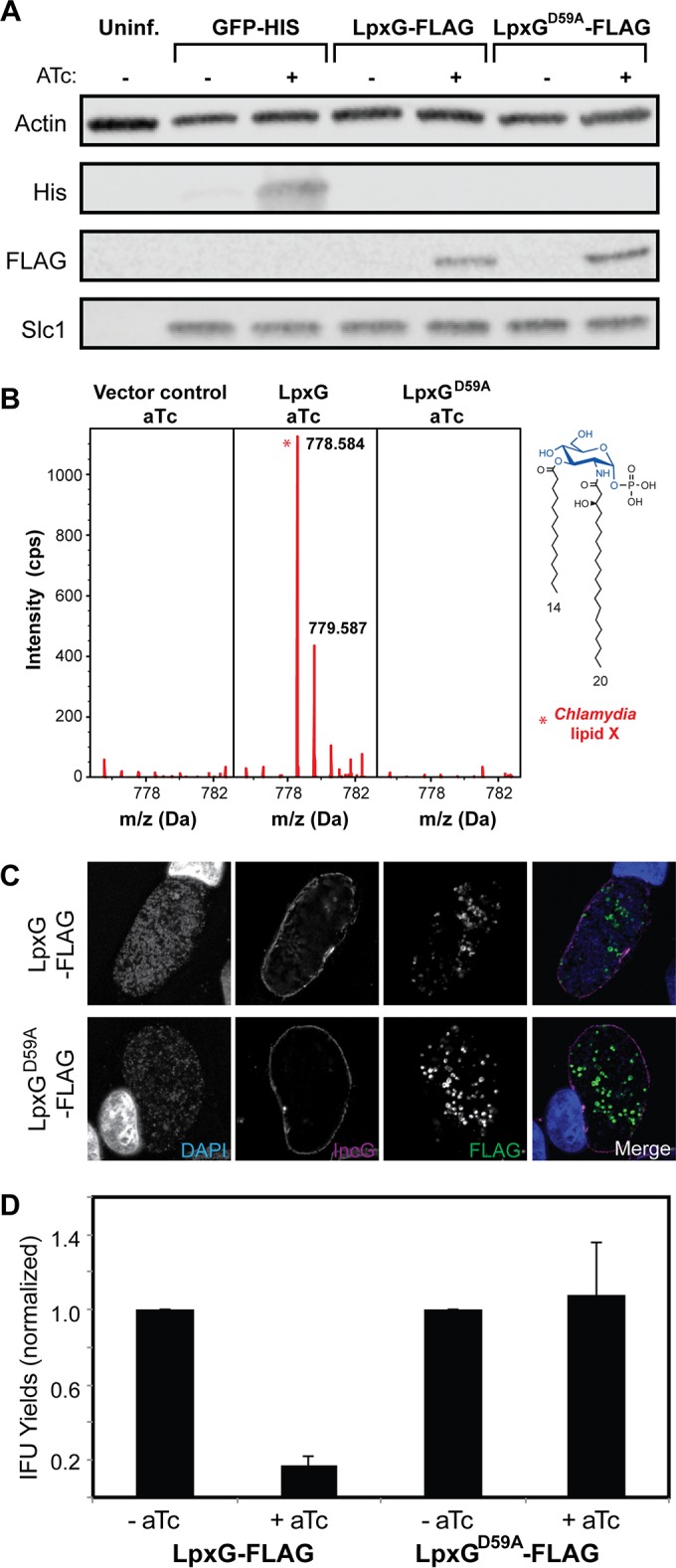
Overexpression of LpxG in *C. trachomatis* leads to lowered infectivity. (A) Inducible expression of *Chlamydia* LpxG. Vero cells were infected with *C. trachomatis* LGV-L2 expressing His_6_-tagged GFP, LpxG-FLAG, or LpxG^D59A^-FLAG under the control of a promoter that is inducible with anhydrous tetracycline (ATc). ATc was added at 16 h postinfection, and total proteins harvested at 30 h. Anti-His_6_ and -FLAG antibodies were used to detect inducible proteins. Anti-actin and -Slc1 antibodies were used to detect host and bacterial proteins, respectively. (B) Mass spectrometry analysis of lipid X in Vero cells infected with *C. trachomatis* transformed with plasmids overexpressing LpxG or the LpxG^D59A^ mutant. *C. trachomatis* transformed with an empty vector was included as a negative control. (C) Subcellular localization of LpxG in *C. trachomatis*. Vero cells were infected as described for panel A and processed for indirect immunofluorescence (IF) microscopy. Bacterial and host DNA were detected with DAPI, the inclusion membrane with anti-IncG antibodies, and LpxG variants with anti-FLAG antibodies. (D) LpxG overexpression leads to lower *Chlamydia* infectivity. HeLa cells were infected as described for panel A, and EBs harvested by hypotonic lysis. The number of EBs was enumerated on Vero cells, and the number of inclusion-forming units (IFUs) assessed by IF microscopy. IFU yields are shown normalized to the yields for noninduced controls. Means and standard deviations are shown for at least three biological replicates.

*C. trachomatis* lymphogranuloma venereum (LGV) strain L2 (LGV-L2) was transformed with an LpxG overexpression vector as described previously (16). The plasmid contained the FLAG-tagged *lpxG* gene under the control of an anhydrous tetracycline (ATc)-inducible promoter, and the expression of LpxG was confirmed by immunoblot analysis with the anti-FLAG antibody (Fig. 6A). ATc was added at 16 h postinfection (hpi) to induce the overexpression of LpxG. For the analysis of lipids, Vero cells infected with strain LGV-L2 containing the LpxG expression vector were lysed and their lipids extracted by the Bligh-Dyer method (14, 17). The relative abundance of the lipids was examined by normal phase LC-ESI/MS operated in the negative ion mode. Significant accumulation of lipid X with a [M−H]^−^ ion at *m*/*z* 778.584 (predicted *m*/*z* 778.524) was observed in the LpxG overexpression cells compared to the vector control cells or cells overexpressing the inactive LpxG^D59A^ mutant (Fig. 6B), confirming that LpxG participates in lipid A biosynthesis in *Chlamydia*. Importantly, the localization of LpxG and the LpxG^D59A^ mutant within infected cells was indistinguishable (Fig. 6C), indicating that the LpxG^D59A^ mutation did not alter the stability of LpxG or its localization to bacterial membranes. ATc-induced overexpression of LpxG but not of the LpxG^D59A^ mutant also resulted in a significant reduction in the generation of infectious *Chlamydia* EBs (Fig. 6D), a phenotype similar to what had been reported with LOS inhibitors (18). Overall, these findings highlight the importance of maintaining controlled lipid A biosynthesis and membrane integrity for the ability of *Chlamydia* to infect cells.

## DISCUSSION

*C. trachomatis* is a leading cause of infectious blindness and the most prevalent sexually transmitted bacterial infection (1, 2, 19). Despite the clinical importance of *Chlamydia*, a functional characterization of its gene products has been hampered by a lack of robust genetic tools, leaving many biologically important activities unresolved, including the elusive gene encoding the UDP-DAGn pyrophosphatase activity that is essential for lipid A biosynthesis in this bacterium. Our studies reinforce the notion that, despite the phylogenetic differences between *C. trachomatis* and the model organism *E. coli*, these bacteria share similar molecular signatures of metabolic and biochemical needs, making it possible to use *E. coli* as a heterologous host to characterize *Chlamydia* gene products (20–22). The screening strategy of using a *Chlamydia* genomic library for heterologous expression in *E. coli* provides an efficient method to identify other *C. trachomatis* genes with unknown functions, especially those that complement essential cellular processes in Gram-negative bacteria.

Such an approach is exemplified by our identification of the *Chlamydia* UDP-DAGn pyrophosphatase, LpxG. LpxG shares extremely low sequence identity with either LpxH (11%) or LpxI (9%), making it impossible to identify LpxG solely based on sequence conservation and highlighting LpxG as the founding member of a third family of UDP-DAGn pyrophosphatases. Importantly, overexpression of LpxG results in the accumulation of lipid X in *Chlamydia* and leads to a decrease of bacterial infectivity. This observation, together with our previous report of a stalled *Chlamydia* infectious cycle when lipid A biosynthesis is blocked by pharmacological inhibition of LpxC (18), emphasizes the functional importance of maintaining balanced lipid A biosynthesis to generate infectious EBs. Given the crucial role of an intact outer membrane environment for the proper functionality of *Chlamydia* outer membrane proteins and secretion systems, it is likely that there exists not only a regulatory mechanism influencing the rate of lipid A biosynthesis but also a lipid A sensing and signaling pathway orchestrating the life cycle of *Chlamydia* infection.

The discovery of LpxG as a UDP-DAGn pyrophosphatase that is distinct from LpxH in beta- and gammaproteobacteria and LpxI in alphaproteobacteria represents a major step forward in our understanding of the lipid A biosynthetic pathway in Gram-negative bacteria. Recently, a potent small-molecule inhibitor of *E. coli* LpxH has been discovered through high-throughput screening (23). The exceedingly low sequence identity of the unique UDP-DAGn pyrophosphatase LpxG compared with LpxH and LpxI forecasts distinct structural features within LpxG that could be exploited for developing highly specific antibiotics for treating *Chlamydia* infections without causing major alterations in resident microbial communities or leading to unintended antibiotic resistance among other coinfecting pathogens.

## MATERIALS AND METHODS

### Chemicals and reagents.

Common chemicals were purchased from Sigma-Aldrich (St. Louis, MO) or from EMD Science (Gibbstown, NJ). Radioactive [^32^P]phosphoric acid was purchased from PerkinElmer (Waltham, MA). Protein concentrations were determined by either the bicinchoninic acid (BCA) assay or the Bradford assay (Thermo Scientific, Waltham, MA). QIAprep spin miniprep kits and QIAquick gel extraction kits (Qiagen, Valencia, CA) were used for plasmid purification and DNA extraction from the agarose gel.

### Plasmids, bacterial strains, and growth conditions.

The plasmids and bacterial strains used in this study are listed in Table S1 and S2, respectively, in the supplemental material (24, 25). P1*vir* phage lysate preparation and infections were carried out following standard procedures (26). Luria-Bertani (LB) broth (Difco, Detroit, MI) was used as the growth medium for liquid culture, and LB broth supplemented with 15 g/liter Bacto agar was used for solid-phase growth. Antibiotics were used at the following concentrations: 100 µg/ml ampicillin (Amp), 50 µg/ml kanamycin (Kan), 25 µg/ml chloramphenicol (Cam).

### Construction of pDEST *C. trachomatis* genomic library.

Each clone from the *C. trachomatis* ORF library harbored in *E. coli* (7) was inoculated into a single well of a microtiter plate containing 200 µl of LB supplemented with Kan and incubated for 18 h at 37°C. The overnight cultures were pooled, and plasmids were extracted to yield the plasmid library pDONR_Ctlib. Gateway technology was employed to transfer the ORF inserts from pDONR_CtLib into pDEST17 to generate pDEST_CtLib. Gateway LR Clonase (Invitrogen, Carlsbad, CA) reactions were carried out according to specifications from the manufacturer, except that reactions were performed at 25°C for 24 h. Aliquots from these reactions were transformed into *E. coli* C41(DE3) cells and plated on LB agar supplemented with Amp. To ensure at least a 20-fold coverage of the entire library, more than 20,000 colonies were collected and pooled, followed by outgrowth for 2 h at 37°C in LB. The final cell sample containing pDEST_CtLib was termed C41(DE3)_CtLib.

### Complementation screen.

A P1*vir* lysate was generated from an Δ*lpxH*::*kan E. coli* strain harboring *lpxH* on the pBAD33 vector (strain W3110AΔHEc) (see Table S2 in the supplemental material) and used to transduce *E. coli* C41(DE3) harboring *lpxH* on the pET21a vector [C41(DE3)EcH] (see Table S2). Transductants were selected on LB-agar plates containing Kan and Amp. The chromosomal deletion of the *lpxH* gene was confirmed by sequencing, and this strain was designated C41(DE3)ΔHEc (see Table S2).

To generate a controllable *lpxH* expression strain, *E. coli* lpxH on a temperature-sensitive plasmid (pKJB5) was transformed into *E. coli* BL21(DE3) cells, and the *ΔlpxH*::*kan* cassette from strain C41(DE3)ΔHEc was transduced by P1*vir* transduction at 30°C, the permissive temperature for the plasmid pKJB5. The resulting strain, HY1 (see Table S2 in the supplemental material), harbored a deletion of *lpxH*, as confirmed by sequencing.

To screen the *C. trachomatis* ORFome for gene(s) that complemented a temperature-mediated *lpxH* disruption, pDEST_CtLib was transformed into strain HY1 and incubated at 44°C. A portion of the transformed cells was incubated at 30°C to determine the transformation efficiency. The pKJB2 plasmid encoding *E. coli lpxH* and an empty pET16b plasmid were also transformed into HY1 as positive and negative controls, respectively. The number of colonies was scored and compared to the numbers in the controls.

### Molecular biology methods.

To generate an expression vector of LpxG with a C-terminal His_10_ tag that was cleavable by tobacco etch virus (TEV) protease, QuikChange (Stratagene, La Jolla, CA) mutagenesis was used to insert additional nucleotides into pET21b (Novagen) to encode the TEV protease recognition site (ENLYFQG) (27), as well as four additional histidine residues needed to elongate the affinity tag. The resulting plasmid, pHSC, was confirmed by sequencing.

*Ct461/lpxG* from the original pDONR library lacks 21 residues of the N-terminal transmembrane helix. The gene encoding full-length LpxG was generated by megaprimer PCR to extend the N-terminal missing residues and then cloned into pHSC to yield LpxG followed by the TEV site and His_10_ tag (pLpxGt). The LpxG^D59A^ mutant was generated by point mutagenesis (pLpxGt_D59A). The presence of the correct sequence was confirmed by DNA sequencing.

In order to verify the UDP-DAGn hydrolase activity of LpxG, W3110A cells harboring either full-length Ct461 in a pBAD33 plasmid (Invitrogen) or an empty vector were transduced with a P1*vir* lysate prepared from W3110AΔHEc, following standard protocols, to replace the chromosomal *lpxH* with a *kan* cassette. Colonies containing the *ΔlpxH*::*kan* insertion can only be isolated from the cells harboring *lpxG* on the plasmid and not from cells containing an empty vector, confirming the functional complementation of LpxH in *E. coli* by full-length LpxG from *C. trachomatis.*

### Protein expression and purification.

Plasmids encoding full-length LpxG (pLpxGt), the LpxG^D59A^ mutant (pLpxGt_D59A), or an empty vector (pET21t10) were transformed into *E. coli* C41(DE3) cells for protein expression. The cells (LpxG_t10, LpxG^D59A^_t10, and VC_t10) were grown at 30°C in LB supplemented with Amp until the optical density at 600 nm (OD_600_) reached 0.7 to 0.8 and then induced with 1 mM IPTG for protein expression for 4 to 5 h. Cells were collected by centrifugation, resuspended in an ice-cold buffer containing 20 mM HEPES, pH 8.0, and passed twice through a French pressure cell (SIM-Aminco; Spectronic Instruments) at 18,000 lb/in^2^. The cell debris was removed by centrifugation at 10,000 × *g*, and the supernatant was collected as cell-free extracts (CFEs) and stored at −80°C.

For protein purification, LpxG was extracted from diluted CFE (5 mg/ml in 300 mM NaCl, 10% glycerol, 20 mM HEPES, pH 8.0) with dodecyl-β-d-maltoside (DDM; Avanti Polar Lipids, Alabaster, AL) at a final detergent concentration of 1% (wt/vol). The detergent-solubilized LpxG was subjected to ultracentrifugation at 100,000 × *g* for 45 min, and the supernatant was purified by Ni-NTA affinity chromatography in the presence of 0.01% DDM. The final protein concentration ranged from 0.15 to 0.5 mg/ml.

### UDP-DAGn hydrolase activity assay.

Autoradiographic assays for the hydrolase activity were similar to that previously described (5, 13), but with slight modifications. The reaction mixtures were a final volume of 12.5 µl in 0.6-ml polypropylene tubes and contained 20 mM HEPES, pH 8.0, 0.5% (wt/vol) BSA, 0.035% (wt/vol) DDM, 1 mM MnCl_2_, 100 µM UDP-DAGn (prepared as previously described [28]), 1,000 cpm/µl [β-^32^P]UDP-DAGn (prepared as previously described [13]), and protein or lysate sample. All reaction mixture components besides the protein sample were mixed to a volume of 10 µl and equilibrated at 30°C for 10 min, after which 2.5 µl of protein was added to start the reaction. The final protein concentrations in the assays ranged from 0.05 mg/ml to 1.0 mg/ml to maintain linear activity within the time frame being tested. Aliquots were taken from the reaction mixtures at various time intervals and spotted onto glass-backed silica gel thin-layer chromatography (TLC) plates (EMD Chemicals, Darmstadt, Germany). These plates were developed in a chloroform-methanol-water-acetic acid (25:15:4:2) tank system, and the data analyzed using PhosphorImager (GE Healthcare) as previously described (5, 13).

### Metal dependence of LpxG.

To analyze the metal dependence of LpxG, a modified version of the autoradiographic assay described above was employed. First, a concentrated stock of EDTA was added to a sample of LpxG to obtain a final chelator concentration of 50 µM EDTA; the protein was then incubated on ice for 30 min. Next, the sample was diluted 10-fold into various reaction mixtures similar to those described above, except that 1 mM MnCl_2_ was replaced with one of the following chloride salts at 1 mM: Ca^2+^, Co^2+^, Cu^2+^, Fe^3+^, Mg^2+^, Mn^2+^, Ni^2+^, and Zn^2+^. Conditions in which the replacing component was 2 mM NaCl or 1 mM EDTA were also included as controls.

### Analysis of LpxG reactions by mass spectrometry.

The lipid products of the LpxG reaction were analyzed by mass spectrometry, employing a method similar to that used for analysis of the LpxI reaction products (6). Briefly, 50-µl reaction mixtures consisting of 100 µM UDP-DAGn, 1 mM MnCl_2_, 20 mM HEPES (pH 8.0), and 0.06 mg/ml LpxG were prepared in the presence of either 100% H_2_^16^O or an H_2_^18^O-H_2_^16^O mixture (70:30, vol/vol) (Sigma-Aldrich, St. Louis MO). After incubation for 2 h at 30°C, the reactions were quenched by conversion to a 1.9-ml single-phase, acidic Bligh-Dyer system (14). A 10-µl aliquot of this material was analyzed by reverse-phase LC–MS using a Shimadzu LC system coupled to a TripleTOF 5600 quadrupole time-of-flight tandem mass spectrometer (AB Sciex, Framingham, MA). The MS instrumental settings for negative ion ESI and tandem MS (MS/MS) analysis of lipid species were as follows: ion spray voltage (IS), −4,500 V; current gas (CUR), 20 lb/in^2^ (pressure); gas 1 (GS1), 20 lb/in^2^; declustering potential (DP), −55 V; and focusing potential (FP), −150 V. Data analysis was performed using Analyst TF1.5 software. LC was operated at a flow rate of 200 µl/min with a linear gradient as follows: 100% mobile phase A was held isocratically for 2 min and then linearly increased to 100% mobile phase B over 14 min and held at 100% B for 4 min. Mobile phase A consisted of methanol–acetonitrile–aqueous 1 mM ammonium acetate (60:20:20, vol/vol/vol). Mobile phase B consisted of 100% ethanol containing 1 mM ammonium acetate. A Zorbax SB-C_8_ reverse-phase column (5-µm particle size and dimensions of 2.1 by 50 mm) was obtained from Agilent.

### Overexpression of LpxG in *C. trachomatis.*

*C. trachomatis* lymphogranuloma venereum (LGV) strain L2 was transformed with either pASK-GFP-L2 (29) or derivatives containing LpxG-FLAG or LpxG^D59A^-FLAG in place of green fluorescent protein (GFP) using previously described transformation methods (30). Vero cells were infected at a multiplicity of infection (MOI) of 3 with the various recombinant *Chlamydia* strains. Plates were spun at 3,000 rpm for 30 min at 10°C to synchronize infections. In all assays, cells were treated with 2 ng/ml ATc at 20 hpi. Samples were collected for Western blot analysis, lipid extraction, immunofluorescence, and determination of infectious progeny at specified time points.

For immunoblot analysis, cell extracts were normalized for total protein content by the Bradford assay, and equal amounts of protein were resolved by SDS-PAGE and transferred to 0.45-µm nitrocellulose membranes. Proteins were detected by incubation of membranes with the antibodies indicated above, followed by fluorescently labeled secondary antibodies. The fluorescence signal was measured using the LI-COR Odyssey imaging system (LI-COR Biosciences).

For the immunofluorescence assay, Vero cells were seeded onto glass coverslips placed in a 24-well plate. At 30 hpi, the coverslips were fixed with 3% formaldehyde–0.025% glutaraldehyde at room temperature for 20 min. The cells were permeabilized with 0.2% Triton X-100 in phosphate-buffered saline (PBS), blocked with 3% bovine serum albumin (BSA) in PBS for 30 min, and stained with antibodies against the inclusion membrane protein IncG (31) and the FLAG epitope (F3165; Sigma). DAPI (4′,6-diamidino-2-phenylindole) was used for staining the nucleus. Confocal images were acquired using a Zeiss LSM 510 inverted confocal microscope.

### Mass spectrometry analysis of lipid X of *C. trachomatis.*

For lipid extraction, Vero cells were seeded into 6-well plates, with 4 plates per sample. At 40 hpi, samples were washed briefly with H_2_O and lysed with 200 µl H_2_O per well. Each sample was pooled, and bacteria stabilized in a sucrose-phosphate-glutamate (SPG) buffer (8 mM Na_2_HPO_4_, 2 mM NaH_2_PO_4_, 220 mM sucrose, 0.50 mM l-glutamic acid). Samples were stored at −80°C until analyzed by mass spectrometry. Lipid X was extracted from the *C. trachomatis* sample by the acidic Bligh-Dyer method as described previously (14, 17). The lipid samples were analyzed by normal-phase LC-MS. Normal-phase LC was performed on an Agilent 1200 quaternary LC system equipped with an Ascentis silica high-performance liquid chromatography (HPLC) column (5-µm particle size and dimensions of 25 cm by 2.1 mm; Sigma-Aldrich, St. Louis, MO). Mobile phase A consisted of chloroform-methanol-aqueous ammonium hydroxide (800:195:5, vol/vol); mobile phase B consisted of chloroform-methanol-water-aqueous ammonium hydroxide (600:340:50:5, vol/vol); and mobile phase C consisted of chloroform-methanol-water-aqueous ammonium hydroxide (450:450:95:5, vol/vol). The elution program consisted of the following: 100% mobile phase A was held isocratically for 2 min and then linearly increased to 100% mobile phase B over 14 min and held at 100% B for 11 min. The LC gradient was then changed to 100% mobile phase C over 3 min, held at 100% C for 3 min, and finally returned to 100% A over 0.5 min and held at 100% A for 5 min. The LC eluent (with a total flow rate of 300 µl/min) was introduced into the ESI source of a high-resolution TripleTOF 5600 mass spectrometer with instrumental settings as described above.

### Infectivity of *C. trachomatis* overexpressing LpxG.

Vero cells were seeded in 96-well plates and infected with various *C. trachomatis* strains. At 40 hpi, cells were subjected to hypotonic lysis by adding 160 µl of H_2_O for 10 min at room temperature, followed by the addition of 40 µl of 5× SPG buffer and storage at −80°C. To determine the numbers of infectious particles, serial dilutions of the lysates were used to infect new monolayers of Vero cells. At 32 hpi, cells were washed with PBS and fixed with methanol for 20 min at room temperature. Samples were blocked with 1% BSA in PBS for 1 h and probed with rabbit anti-Slc1 antibodies (30), followed by secondary Alexa Fluor 555-labeled goat anti-rabbit antibodies (Thermo Fisher). Inclusions were counted on a Cellomics ArrayScan high-content imaging system (Thermo Fisher).

## SUPPLEMENTAL MATERIAL

Table S1  Plasmids used in the discovery of LpxG. Table S1, DOC file, 0.03 MB

Table S2  Strains used in the discovery of LpxG. Table S2, DOCX file, 0.03 MB
