# Supplementary material for: Discovery of the Elusive UDP-Diacylglucosamine Hydrolase in the Lipid A Biosynthetic Pathway in Chlamydia trachomatis
Source: mBio. 2016 Mar 22;7(2):e00090-16. doi: 10.1128/mBio.00090-16 (PMC4807358; doi:10.1128/mBio.00090-16)
Supplement: Table S1 — Plasmids used in the discovery of LpxG. [file mbo002162731st1.doc]

Table S1. Plasmids Used in the Discovery of LpxG

| Plasmid | Description | Source or Reference |
| --- | --- | --- |
| pKJB5 | pMAK705 harboring the *E. coli* gene of LpxH; Cam^r^ | (24, 25) |
| pDONR221 | Gateway® entry vector; Kan^r^ | Invitrogen |
| pDONR_CtLib | pDONR221 harboring *C. trachomatis* ORFome library*;* Kan^r^ | This work |
| pDEST17 | Gateway® destination vector compatible with T7 expression; encodes for N-terminal His_6_ fusion; contains ribosome binding site and ATG start codon upstream His-tag; Amp^r^ | Invitrogen |
| pDEST_CtLib | pDEST17 containing *C. trachomatis* ORFome library; Amp^r^ | This work |
| pET21t10 | Modified pET21b with TEV-protease cleavage and His_10_ tag 3' of *XhoI* site; Amp^r^ | (13) |
| pHSC | Modified pET21b with TEV-protease cleavage site and His_10_ tag 3' of HindIII site; Amp^r^ | This work |
| pKJB2 | pET21a^+^ plasmid containing the *E. coli* gene of LpxH*;* Amp^r^ | (5) |
| pLpxGt | pHSC harboring *C. trachomatis* ORF 461 encoding LpxG lacking a stop codon to generate a C-terminal His_10_ tag cleavable by TEV-protease; Amp^r^ | This work |
| pLpxGt_D59A | pLpxGt with LpxG^D59A^ mutation; Amp^r^ | This work |
