# Supplementary material for: Discovery of the Elusive UDP-Diacylglucosamine Hydrolase in the Lipid A Biosynthetic Pathway in Chlamydia trachomatis
Source: mBio. 2016 Mar 22;7(2):e00090-16. doi: 10.1128/mBio.00090-16 (PMC4807358; doi:10.1128/mBio.00090-16)
Supplement: Table S2 — Strains used in the discovery of LpxG. [file mbo002162731st2.docx]

Table S2. Strains Used in the Discovery of LpxG

| Strain | Description | Source or Reference |
| --- | --- | --- |
| C41(DE3)_CtLib | C41(DE3), pDEST_Ctlib | This work |
| C41(DE3)Ec | C41(DE3), pKJB2 | This work |
| C41(DE3)ΔHCtG | C41(DE3) ΔlpxH::kan, pKJB2 | This work |
| HY1 | BL21(DE3) *ΔlpxH::kan,* pKJB5 | This work |
| HY1_Lib | BL21(DE3) *ΔlpxH::kan,* pKJB5, pDEST17_CtLib | This work |
| HY1_VC | BL21(DE3) *ΔlpxH::kan,* pKJB5, pHSC | This work |
| LpxG_t10 | C41(DE3) harboring pLpxGt | This work |
| VC_t10 | C41(DE3) harboring an empty modified pET21 vector (pET21t10) | ([13](#_ENREF_13)) |
| LpxG^D59A^_t10 | C41(DE3) harboring LpxG D59A on a pET21 vector (pLpxGt_D59A) | This work |
| W3110AΔHEc | W3110A *lpxH::kan* harboring pBAD33EcLpxH | ([6](#_ENREF_6)) |
